# The prognostic value of T1 mapping and late gadolinium enhancement cardiovascular magnetic resonance imaging in patients with light chain amyloidosis

**DOI:** 10.1186/s12968-017-0419-6

**Published:** 2018-01-03

**Authors:** Lu Lin, Xiao Li, Jun Feng, Kai-ni Shen, Zhuang Tian, Jian Sun, Yue-ying Mao, Jian Cao, Zheng-yu Jin, Jian Li, Joseph B. Selvanayagam, Yi-ning Wang

**Affiliations:** 1 Department of Radiology, Peking Union Medical College Hospital, Chinese Academy of Medical Sciences & Peking Union Medical College, No.1, Shuaifuyuan, Dongcheng District, Beijing, 100730 China; 2 Department of Hematology, Peking Union Medical College Hospital, Chinese Academy of Medical Sciences & Peking Union Medical College, No.1, Shuaifuyuan, Dongcheng District, Beijing, 100730 China; 3 Department of Cardiology, Peking Union Medical College Hospital, Chinese Academy of Medical Sciences & Peking Union Medical College, No.1, Shuaifuyuan, Dongcheng District, Beijing, 100730 China; 4 Department of Pathology, Peking Union Medical College Hospital, Chinese Academy of Medical Sciences & Peking Union Medical College, No.1, Shuaifuyuan, Dongcheng District, Beijing, 100730 China; 5 Department of Cardiovascular Medicine, Flinders University, Flinders Medical Centre, Bedford Park, Adelaide, 5042 SA Australia

**Keywords:** Light chain amyloidosis, Cardiovascular magnetic resonance imaging, T1 mapping, Late gadolinium enhancement

## Abstract

**Background:**

Cardiac impairment is associated with high morbidity and mortality in immunoglobulin light chain (AL) type amyloidosis, for which early identification and risk stratification is vital. For myocardial tissue characterization, late gadolinium enhancement (LGE) is a classic and most commonly performed cardiovascular magnetic resonance (CMR) parameter. T1 mapping with native T1 and extracellular volume (ECV) are recently developed quantitative parameters. We aimed to investigate the prognostic value of native T1, ECV and LGE in patients with AL amyloidosis.

**Methods:**

Eighty-two patients (55.5 ± 8.5 years; 52 M) and 20 healthy subjects (53.2 ± 11.7 years; 10 M) were prospectively recruited. All subjects underwent CMR with LGE imaging and T1 mapping using a Modified Look-Locker Inversion-recovery (MOLLI) sequence on a 3 T scanner. Native T1 and ECV were measured semi-automatically using a dedicated CMR software. The left ventricular (LV) LGE pattern was classified as none, patchy, and global groups. Global LGE was considered when there was diffuse, transmural LGE in more than half of the short axis images. Follow-up was performed for all-cause mortality using Cox proportional hazards regression analysis and Kaplan-Meier survival curves.

**Results:**

The patients demonstrated an increase in native T1 (1438 ± 120 ms vs. 1283 ± 46 ms, *P* = 0.001) and ECV (43.9 ± 10.9% vs. 27.0 ± 1.7%, *P* = 0.001) compared to healthy controls. Native T1, ECV and LGE showed significant correlation with Mayo Stage, and ECV and LGE showed significant correlation with echocardiographic E/E’ and LV ejection fraction. During the follow-up for a median time of 8 months, 21 deaths occurred. ECV ≥ 44.0% (hazard ratio [HR] 7.249, 95% confidence interval (CI) 1.751–13.179, *P* = 0.002) and global LGE (HR 4.804, 95% CI 1.971–12.926, *P* = 0.001) were independently prognostic for mortality over other clinical and imaging parameters. In subgroups with the same LGE pattern, ECV ≥ 44.0% remained prognostic (log rank *P* = 0.029). Median native T1 (1456 ms) was not prognostic for mortality (Tarone-Ware, *P* = 0.069).

**Conclusions:**

During a short-term follow-up, both ECV and LGE are independently prognostic for mortality in AL amyloidosis. In patients with a similar LGE pattern, ECV remained prognostic. Native T1 was not found to be a prognostic factor.

## Background

Immunoglobulin light chain (AL) type amyloidosis is characterized by monoclonal plasma cells and the deposition of insoluble fibrils formed by immunoglobulin light chains in various organs [1]. In approximately two-thirds of AL-type amyloidosis patients there is cardiac impairment at diagnosis, which is a major contributor to mortality [2]. Thus, early identification and risk stratification is of vital importance for timely clinical intervention that may improve the patients’ prognosis. Current predictors of survival, such as serum biomarkers [3–5], electrocardiogram (ECG) [6], cardiac morphology and functional parameters [7–10] rely on measuring surrogates rather than direct markers of interstitial expansion.

Cardiovascular magnetic resonance (CMR) imaging with late gadolinium enhancement (LGE) is the most commonly performed non-invasive protocol for myocardial tissue characterization in a wide spectrum of cardiomyopathies. A typical pattern of global, predominately subendocardial LGE, serves not only as a diagnostic marker for cardiac AL amyloidosis but also as a prognostic marker for mortality [11–14]. However, because the recognition of LGE lesions involves delineation of abnormal tissue from normal tissue, early identification of mild cases can easily be missed in cardiac AL amyloidosis and other diffuse infiltrative cardiomyopathies [15–17].

Myocardial CMR T1 mapping methods are used for native (i.e., without use of gadolinium-based agents) and for post-contrast T1 measurements. In combination with the hematocrit, pre- and post-contrast measurements enable the quantification of the extracellular volume fraction (ECV). Native myocardial T1 values reflect a composite signal from both the intracellular (predominantly myocytes) and extracellular compartments [18–20]. Previous studies have shown that different sequences and field strengths yielded different native T1 and ECV values [21–24]. To date, only one study has examined the utility of a shortened Modified Look-Lockers Inversion-recovery (shMOLLI) sequence at 1.5 T to assess the prognostic value of native T1 and ECV in AL amyloidosis [25]. However, this study did not concurrently assess the utility of LGE in this population. In the present study, we examined a Chinese population with AL amyloidosis using a 3 T scanner with a MOLLI sequence and compared the prognostic value of T1 mapping parameters with LGE. This method of analysis of the prognostic values of native T1 and ECV for mortality in AL amyloidosis and its comparison with LGE have not been reported previously.

## Methods

### Study subjects

This prospective study was approved by the Institutional Ethnics Committee for Human Research at Peking Union Medical College Hospital (Beijing, China). All participants were required to provide written informed consent prior to recruitment. AL amyloidosis patients who were referred for CMR imaging at Peking Union Medical College Hospital between August 1, 2014 and August 31, 2016 were included in the study. Approximately 20% of the patients who had contraindications either to CMR imaging (i.e., CMR-incompatible devices) or contrast administration (i.e., estimated glomerular filtration rate < 30 ml/min) were excluded.

Eighty-two AL amyloidosis patients (55.5 ± 8.5 years; 52 male) were consecutively recruited. All patients had biopsy evidence of AL amyloidosis with positive Congo red stain and light chain deposition confirmed by immunohistochemistry, immunofluorescence or mass spectrometer. The assays were performed in the tissues listed as follows: kidney (*n* = 29), myocardium (*n* = 19), bone marrow (*n* = 7), fat (n = 7), tongue (n = 7), liver (*n* = 4), upper gastrointestinal tract (*n* = 3), buccal mucosa (n = 3), lung (n = 1), rectum (n = 1) and skin (n = 1). All patients underwent laboratory examination of the cardiac biomarkers Troponin I (cTnI) and N-terminal pro-B-type natriuretic peptide (NT-proBNP), serum immunoglobulin free light chain difference (dFLC) at baseline and were categorized based on revised Mayo Stage published in 2012 [5]. All patients underwent transthoracic echocardiography (TTE) at baseline and the E: E′ ratio and E: A ratio were calculated to assess the left ventricular (LV) diastolic function. A hematologist and a cardiologist, both of whom were blinded to the results of CMR imaging, recorded the results of Mayo Stage and TTE, respectively.

Twenty healthy subjects (53.2 ± 11.7 years; 10 male) with normal CMR imaging results were recruited, who had neither history nor symptoms of cardiovascular disease or diabetes mellitus.

### CMR scanning protocol

CMR was performed on a 3 T whole-body scanner (MAGNETOM Skyra, Siemens Healthineers, Erlangen, Germany). The system is capable of operating at a maximum slew rate of 200 mT/m/ms and a maximum gradient strength of 45 mT/m. An 18-element body matrix coil and a 32-element spine array coil were used for data acquisition. A four-lead vector cardiogram was used for ECG gating.

Two-dimensional (2D) scout images in transversal, coronal and sagittal views were first acquired for localization of the heart. The cine images were acquired with an ECG-gated 2D balanced steady-state free precession (bSSFP) sequence during multiple breath holds. To evaluate cardiac motion and function, two-, three-, and four-chamber long-axis and 10–12 short-axis slices covering the LV were acquired. The key parameters were as follows: repetition time (TR)/echo time (TE), 3.3/1.43 msec; flip angle (FA), 55°–70°; voxel size, 1.6 × 1.6 × 6.0 mm; temporal resolution, 45.6 msec; bandwidth, 962 Hz/pixel. Native and 15–20 min post-contrast T1 mapping were acquired using a MOLLI sequence in identical imaging locations, including a four-chamber long-axis slice and three short-axis slices (apex, mid-ventricular, and basic) [26]. Acquisition schema 5(3)3 and 4(1)3(1)2 were used for pre-contrast and post-contrast T1 mapping, respectively. To generate pixel-wise myocardial T1 maps, single-shot-bSSFP images were acquired at different inversion times and registered prior to a non-linear least-square curve fitting [27, 28]. The other parameters included: TR/TE/flip angle, 2.7 ms/1.12 ms/20°; voxel size, 1.4 × 1.4 × 8.0 mm. LGE images were collected by a 2D phase-sensitive inversion-recovery (PSIR) gradient-echo pulse sequence with breath-hold. Parameters of the sequence were as follows: TR/TE/flip angle, 5.2 ms/1.96 ms/20°; voxel size, 1.4 × 1.4 × 8.0 mm.

### CMR image analysis

CMR images were independently analyzed by two experienced radiologists. The LV LGE pattern was classified into three groups referred to Araoz Criteria [11] and Moon Criteria [12]: No LGE, when there were no areas of LGE; Patchy LGE, when there were discrete areas of LGE, or there were diffuse areas of LGE in less than half of the short axis images; Global LGE, when there was diffuse, transmural LGE in more than half of the short axis images. Discrepancies were resolved in consensus during a joint evaluation with a third radiologist.

Cardiac function, native T1 and ECV were measured semi-automatically using a dedicated CMR software cvi42 (version 5.3, Circle Cardiovascular Imaging, Calgary, Canada). Standard parameters of cardiac structure (i.e., inter-ventricular septum thickness, ventricle volume, LV mass and left atrium area with indexing for body surface area) and ventricle ejection fraction were measured by contouring the endocardium and epicardium on long-axis and short axis cine images at the end-systolic and end-diastolic stage. Native T1 and ECV of the 16 American Heart Association (AHA) segments and global LV were measured, by contouring the endocardium and epicardium and indicating the inter-ventricular septum on pre-contrast and post-contrast T1 mapping images with indexing for the hematocrit. Global LV native T1 and ECV were used for further analysis. The average values of native T1 and ECV measured by the two radiologists were used.

### Clinical follow-up

A physician blinded to the results of CMR imaging conducted the telephone and clinical follow-up each month. Unless the outcome was death from any cause, patients were censored at the end of the study. If patients were lost to follow-up, their last clinic visit record was used. A follow-up CMR scan was performed after a complete standard course of chemotherapy, with an interval of about approximately one year.

### Statistical analysis

Statistical analysis was performed using SPSS Statistics (version 21.0, International Business Machines, Inc., Armonk, New York, USA) and R programming language for statistical computing (version 3.0.1, The R Foundation for Statistical Computing). The agreement between two observers was assessed using the interclass correlation coefficient. Correlation between native T1 and ECV with continuous variables or categorical variables was assessed using the Pearson’s r correlation or Spearman ρ correlation, respectively. Comparison between groups and the control was performed by one-way analysis of variance (ANOVA) with post-hoc Bonferroni correction. Statistical significance was defined as *P* < 0.05.

Survival was evaluated with Cox proportional hazards regression analysis, providing estimated hazard ratios (HR) with 95% confidence intervals (CI) and Kaplan-Meier curves. All variables were first analyzed with univariate Cox regression. Multivariate models were then used to evaluate the independent prognostic value of native T1, ECV or LGE above other clinically and statistically significant covariates. The median value of native T1 and ECV was used as cut-off values. The Harrell’s C statistic was calculated for different models.

## Results

### Baseline characteristics and clinical outcome

Table 1 summarizes the characteristics of AL amyloidosis patients and healthy controls at baseline. At the time of CMR scanning, 9 (11%) patients had received triple chemotherapy for the first time with thalidomide or bortezomib, cyclophosphamide and dexamethasone (BCD or TCD), 2 (2%) had received autologous stem cell transplant (ASCT) and 71 (87%) had not received any chemotherapy. During the follow-up, 59 (83%) untreated patients received standardized treatment with chemotherapy or ASCT, and the rest did not receive any chemotherapy because of the expense or for personal or other reasons. At the time of last follow-up, 61 (74%) patients were alive, with a survival probability of approximately 75.6% at median follow-up time (8 months). Two patients were lost to follow-up. The follow-up time of one patient (female; 52 years; Mayo Stage, III; LVEF, 52.5%; native T1, 1575 ms; ECV, 51.4%; LGE pattern, global) was 5 months, and the other (male; 68 years; Mayo Stage, II; LVEF, 55.5%; native T1, 1512 ms; ECV, 41.6%; LGE pattern, global) was 18 months.

**Table 1:** Baseline characteristics of the AL amyloidosis patients and healthy controls

Characteristics	Patients *n* = 82	Healthy controls *n* = 20	*P*
Clinical			
Male/female	52/30	10/10	0.27
Age (years)	55.5 ± 8.5	53.2 ± 11.7	0.30
NYHA (I/II/III/IV)	30/24/23/5	–	–
cTnI (μg/L)	0.043 (0.015–0.146)	0.000 (0.000–0.040)	0.024
NT-proBNP (pg/mL)	2056 (348–6096)	0 (0–23)	0.001
dFLC (mg/L)	138.0 (46.0–391.5)	–	–
Mayo Stage (I/II/III/IV)	22/18/29/13	–	–
Creatinine (umol/L)	87.3 ± 21.6	74.9 ± 15.3	0.21
HTN/CHD/DM/Af	16/6/3/2	–	–
Therapy (BCD/TCD/ASCT)	5/4/2	–	–
Echocardiography			
E/A	1.3 ± 0.7	–	–
E/E’	16.8 ± 8.3	–	–
Cardiac MR			
Indexed LVEDV (ml/m^2^)	58.3 ± 16.0	74.5 ± 17.1	0.001
Indexed LVESV (ml/m^2^)	22.1 ± 12.4	21.5 ± 8.1	0.79
LVEF (%)	63.3 ± 14.6	70.3 ± 8.7	0.043
Left atrium area (cm^2^)	21.4 ± 5.0	20.6 ± 5.0	0.52
Indexed left ventricle mass (g/m^2^)	93.5 ± 29.0	65.2 ± 15.3	0.001
Septal thickness (mm)	15.4 ± 4.0	10.5 ± 2.0	0.001
LGE (no/patchy/global)	26/18/38	–	–
Native T1 (ms)	1438 ± 120	1283 ± 46	0.001
ECV (%)	43.9 ± 10.9	27.0 ± 1.7	0.001

All continuous variables are presented as mean ± SD, except for cTnI, NT-proBNP and dFLC, which are presented as medians (quartiles 1-quartiles 3). *cTnI* Cardiac Troponin I, *NT-proBNP* N-terminal pro-B-type natriuretic peptide, *dFLC* Serum immunoglobulin free light chain difference, *NYHA* New York Heart Association, *HTN* Hypertension, *CHD* Coronary artery heart disease, *DM* Diabetes mellitus, *Af* Atrial fibrillation, *BCD* Bortezomib, cyclophosphamide and dexamethasone, *TCD* Thalidomide, cyclophosphamide and dexamethasone, *ASCT* Autologous stem cell transplant, *MR* Magnetic resonance, *LVEDV* Left ventricle end-diastolic volume, *LVESV* Left ventricle end-systolic volume, *LVEF* Left ventricle ejection fraction, *LGE* Late gadolinium enhancement, *ECV* Extracellular volume

### Clinical and biochemical markers of severity

All continuous variables were normally distributed (Kolmogorov-Smirnov test) and presented as the mean ± SD, except for cTnI, NT-proBNP and dFLC, which were log transformed for bivariate testing and presented as medians (quartiles 1-quartiles 3). As shown in Tables 1, 30 (37%), 24 (29%), 23 (28%) and 5 (6%) patients were classified under NYHA Classification I, II, III and IV, respectively. Patients showed an increase in cTnI (0.043 [0.015–0.146] μg/L vs. 0.000 [0.000–0.040] μg/L, *P* = 0.024) and NT-proBNP (2056 [348–6096] pg/mL vs. 0 [0–23] pg/mL, *P* = 0.001) compared to healthy controls. There were 22 (27%), 18 (22%), 29 (35%) and 13 (16%) patients in Mayo Stage I, II, III and IV, respectively. Table 2 summarizes the univariate and multivariate Cox proportional hazard analysis of overall survival in all patients. The following were significantly associated with total mortality in univariate analysis: age (HR 1.059, 95% CI 1.008–1.112, *P* = 0.023), NYHA Classification (HR 2.534, 95% CI 1.581–4.062, *P* = 0.001), log (cTnI) (HR 2.568, 95% CI 1.204–5.477, *P* = 0.015), log (NT-proBNP) (HR 3.122, 95% CI 1.501–6.496, *P* = 0.002), Mayo Stage (HR 2.111, 95% CI 1.323–3.368, P = 0.002) and E/E’ (HR 1.089, 95% CI 1.012–1.110, *P* = 0.045).

**Table 2:** Univariate and multivariate Cox proportional hazard analysis in all AL amyloidosis patients

	Univariate		Multivariate		Multivariate	
	HR (95% CI)	*P*	HR (95% CI)	*P*	HR (95% CI)	*P*
Age, per 1 year increase	1.059 (1.008–1.112)	0.023	1.082 (1.022–1.144)	0.006	1.063 (1.000–1.129)	0.051
NYHA	2.534 (1.581–4.062)	0.001	1.569 (0.880–2.797)	0.127	2.253 (1.385–3.666)	0.001
log (cTnI), per unit increase	2.568 (1.204–5.477)	0.015	–	–	–	–
log (NT-proBNP), per unit increase	3.122 (1.501–6.496)	0.002	–	–	–	–
Mayo Stage	2.111 (1.323–3.368)	0.002	1.121 (0.603–2.081)	0.718	1.525 (0.846–2.748)	0.16
E/E’, per 1 unit increase	1.089 (1.012–1.110)	0.045	1.783 (0.334–9.501)	0.498	1.722 (0.318–9.267)	0.43
LVEF, per 1% increase	0.961 (0.936–0.986)	0.003	0.982 (0.948–1.017)	0.307	0.983 (0.951–1.017)	0.33
Septal thickness, per 1 mm increase	1.132 (1.040–1.232)	0.004	1.175 (1.035–1.335)	0.013	1.130 (1.018–1.255)	0.022
ECV ≥44.0%	7.677 (2.256–26.128)	0.001	7.249 (2.039–25.771)	0.002	–	–
Global LGE	5.047 (1.971–12.926)	0.001	–	–	4.804 (1.751–13.179)	0.002

All significantly prognostic factors in univariate analysis were listed. Univariate analysis was not performed for native T1 because the Kaplan-Meier curves crossed each other (Tarone-Ware, *P* = 0.069). All clinically and statistically significant variates in univariate analysis were put into the multivariate Cox model, except for log (cTnI) and log (NT-proBNP), as they were included in Mayo Stage. ECV and LGE were put in separate models because of a correlation ρ of 0.889. Backward regression was chosen

*HR* Hazard ratio, *CI* Confidence interval, *NYHA* New York Heart Association, *cTnI* Cardiac Troponin I, *NT-proBNP* N-terminal pro-B-type natriuretic peptide *LVEF* Left ventricle ejection fraction, *LGE* Late gadolinium enhancement, *ECV* Extracellular volume

### CMR structural and functional parameters

As shown in Tables 1 and 2, AL amyloid patients demonstrated a decrease in LV end-diastolic volume index (LVEDVi) (58.3 ± 16.0 ml/m^2^ vs. 74.5 ± 17.1 ml/m^2^, *P* = 0.001) and LV ejection fraction (LVEF) (63.3 ± 14.6% vs. 70.3 ± 8.7%, *P* = 0.043), as well as an increase in indexed LV mass index (93.5 ± 29.0 g/m^2^ vs. 65.2 ± 15.3 g/m^2^, *P* = 0.001) and inter-ventricular septal thickness (15.4 ± 4.0 mm vs. 10.5 ± 2.0 mm, P = 0.001) compared to healthy controls. Univariate analysis showed that LVEF (HR 0.961, 95% CI 0.936–0.986, *P* = 0.003) and septal thickness (HR 1.132, 95% CI 1.040–1.232, *P* = 0.004) were significant predictors of mortality.

### LGE, native T1 and ECV

**Fig. 1:**
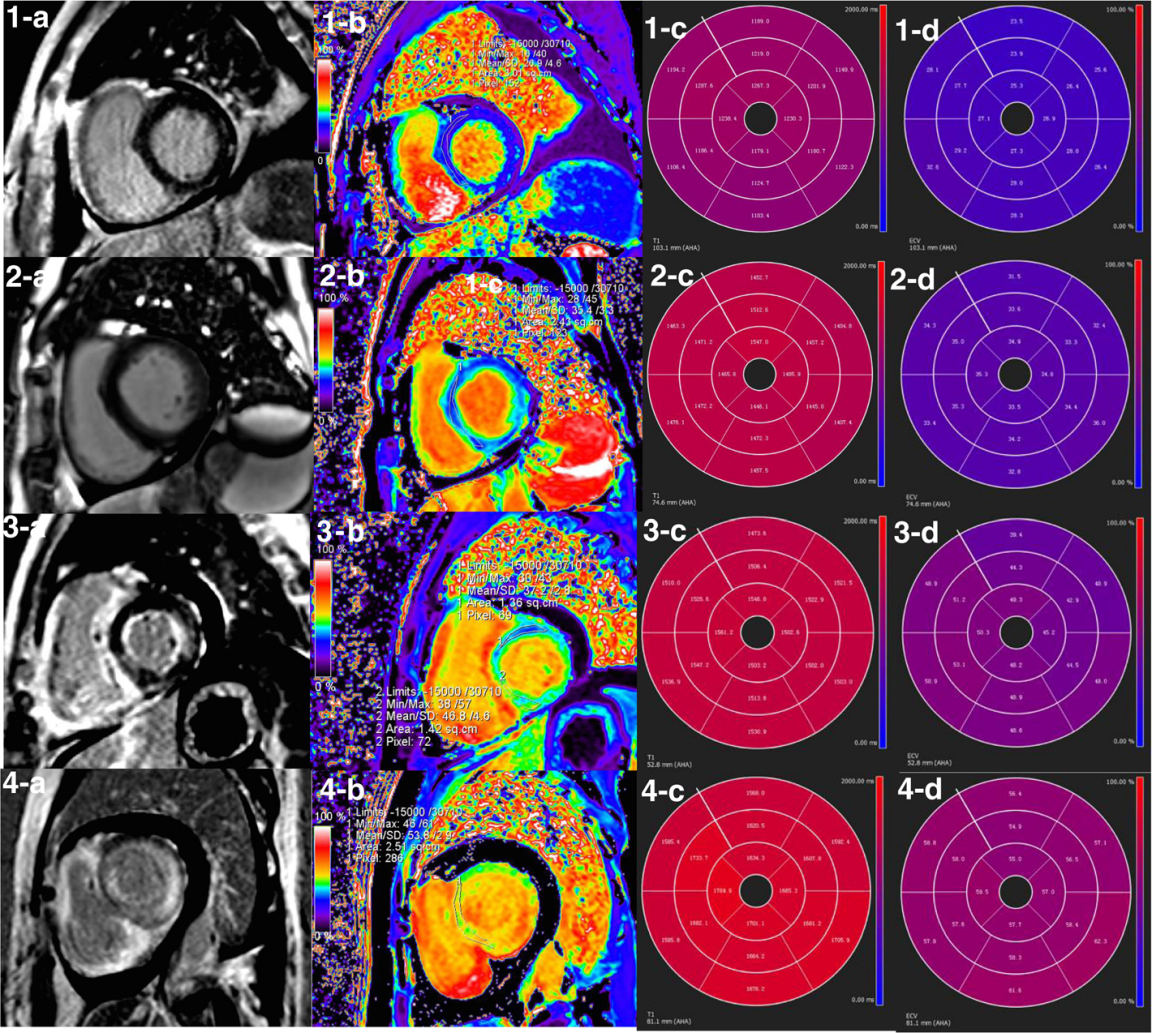
LGE image, ECV pseudo-color image, native T1 and ECV bull’s eye plots of AL amyloid patients and healthy control subjects. (1-a, b, c, d) A healthy control subject displayed no LGE and normal native T1 and ECV at the same slice position. (2-a, b, c, d) A Patient showed no LGE, but increased native T1 and ECV at the same slice position. (3-a, b, c, d) A Patient showed patchy LGE and increased native T1 and ECV at the same slice position, especially in the LGE lesion. (4-a, b, c, d) A Patient showed global LGE and increased native T1 and ECV at the same slice position. LGE = late gadolinium enhancement, ECV = extracellular volume. AHA = American Heart Association

**Fig. 2:**
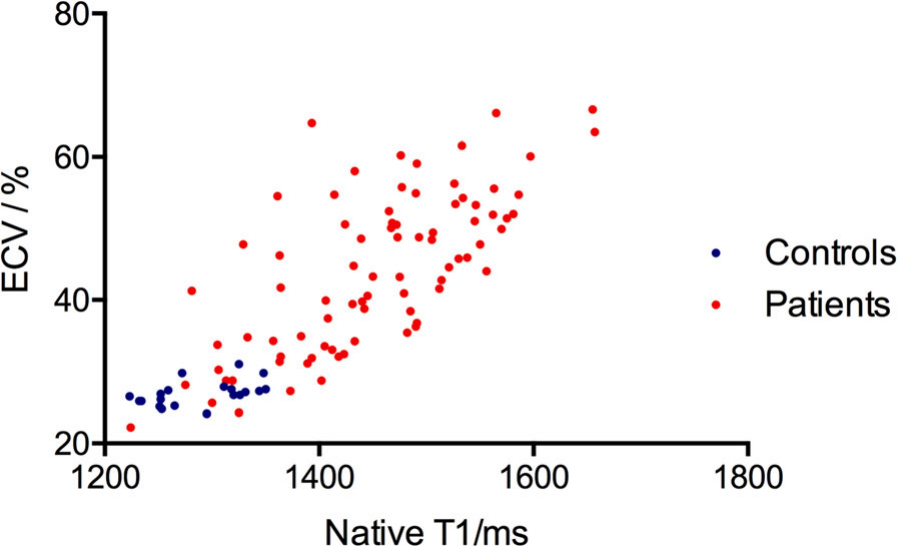
A point spread diagram of the native T1 and ECV values of all AL amyloid patients and heathy subjects. ECV = extracellular volume

**Fig. 3:**
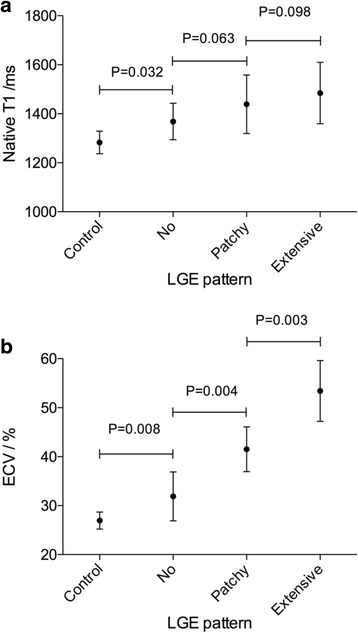
Native T1 and ECV values in AL amyloid subgroups with different LGE patterns. (**a**) Patients with no LGE showed an increase in native T1 (1368 ± 75 ms vs. 1283 ± 46 ms, *P* = 0.032), as compared to healthy controls. (**b**) Patients with no LGE showed an increase in ECV (31.9 ± 5.0% vs. 27.0 ± 1.7%, *P* = 0.008), as compared to healthy controls. LGE = late gadolinium enhancement, ECV = extracellular volume

Representative examples of LGE pattern, native T1 and ECV values from a healthy subjects and AL amyloid patients with different disease burdens are shown in Fig. 1. A point spread diagram of the native T1 and ECV values of all AL amyloidosis patients and healthy subjects are shown in Fig. 2. Patients showed an increase in native T1 (1438 ± 120 ms vs. 1283 ± 46 ms, *P* = 0.001) and ECV (43.9 ± 10.9% vs. 27.0 ± 1.7%, *P* = 0.001) compared to healthy controls. The intra-observer and inter-observer variabilities as well as native T1 reproducibility are shown in Table 3. There were 26 (32%), 18 (22%) and 38 (46%) patients with no LGE, patchy LGE and global LGE, respectively. The Kappa coefficient of classification between the two radiologists was 0.818. The native T1 and ECV values in subgroups with different LGE patterns are shown in Fig. 3. Table 4 summarizes the correlation of native T1, ECV and LGE with clinical, TTE and other CMR parameters in AL amyloid patients. Native T1, ECV and LGE showed significant correlation with each other. Native T1, ECV and LGE showed significant correlation with NYHA classification, NT-proBNP and Mayo Stage, and ECV and LGE showed significant correlation with echocardiographic E/E’.

**Table 3:** T1 mapping intra-observer and inter-observer variabilities and native T1 reproducibility showed by Bland-Altman Plot

	Bias	SD of bias	95% CI
Native T1			
Intra-observer variability 1/ms	−5.60	16.40	−37.04, 25.84
Intra-observer variability 2/ms	4.20	19.74	−34.49, 42.89
Inter-observer variability/ms	6.12	18.33	−32.88, 44.51
Repeated scan reproducibility/ms	5.45	21.07	−35.86, 46.76
ECV			
Intra-observer variability 1/%	0.33	1.44	−2.96, 3.02
Intra-observer variability 2/%	0.27	1.87	−2.41, 3.15
Inter-observer variability 1/%	−0.25	1.82	−3.36, 2.80

Repeated pre-contrast T1 mapping scans were performed within one day on 20 volunteers (native T1, 1283 ± 46 ms; LVEF, 65.4 ± 5.7%) and 20 patients (Mayo Stage I/II/III/IV, n = 4/6/8/2; none/patchy/global LGE, *n* = 6/4/10; native T1, 1498 ± 108 ms; LVEF, 58.6 ± 11.1%). For all patients and volunteers, T1 mapping images were independently analyzed by two experienced radiologists twice. The average value was used. *SD* Standard deviation, *CI* Confidence interval, *ECV* Extracellular volume

**Table 4:** Native T1, ECV and LGE correlation with clinical stages, echocardiographic and other cardiac MR parameters in AL amyloidosis patients

	Native T1		ECV		LGE	
	r or ρ	*P*	r or ρ	*P*	ρ	*P*
Clinical						
NYHA	0.427	0.001	0.686	0.001	0.674	0.001
NT-proBNP	0.351	0.001	0.707	0.001	0.729	0.001
Mayo Stage	0.335	0.002	0.631	0.001	0.671	0.001
Echocardiography						
E/A	0.060	0.65	0.309	0.20	0.330	0.20
E/E’	0.302	0.209	0.488	0.001	0.351	0.006
Cardiac MR						
Indexed LVEDV	−0.222	0.025	−0.320	0.001	−0.113	0.31
Indexed LVESV	−0.078	0.44	0.209	0.036	0.203	0.067
LVEF	0.063	0.93	−0.451	0.001	−0.380	0.001
Left atrium area	0.207	0.037	0.174	0.082	0.197	0.077
Indexed left ventricle mass	0.360	0.001	0.633	0.001	0.590	0.001
Septal thickness	0.440	0.001	0.626	0.001	0.654	0.001
Native T1	–	–	0.605	0.001	–	–
LGE	0.420	0.001	0.867	0.001	–	–

Correlation between native T1 or ECV with continuous variables was assessed using Pearson’s r correlation and with categorical variables using Spearman ρ correlation. Correlation between LGE with other variables was assessed using Spearman ρ correlation

*NYHA* New York Heart Association, *NT-proBNP* N-terminal pro-B-type natriuretic peptide, *MR* Magnetic resonance, *LVEDV* Left ventricle end-diastolic volume, *LVESV* Left ventricle end-systolic volume, *LVEF* Left ventricle ejection fraction, *LGE* Late gadolinium enhancement, *ECV* Extracellular volume

**Fig. 4:**
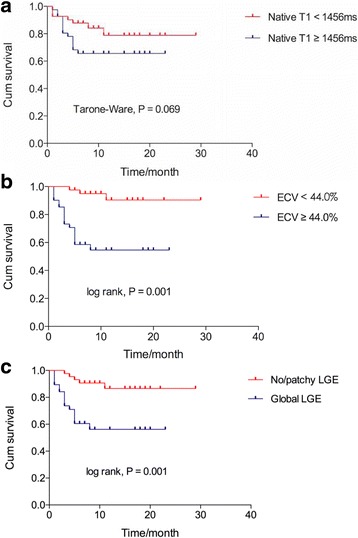
Kaplan-Meier survival curves for native T1, ECV and LGE. (**a**) Patients categorized by median native T1 (1456 ms) did not differ significantly in survival probability (74.1% vs. 65.7% at the 8th month, Tarone-Ware *P* = 0.069). (**b**) Patients with ECV < 44.0% and ECV ≥ 44.0% differed significantly in survival probability (94.9% vs. 54.6% at the 8th month, log rank *P* = 0.001). (**c**) Patients with no/patchy LGE and global LGE differed significantly in survival probability (90.7% vs. 56.2% at the 8th month, log rank P = 0.001). LGE = late gadolinium enhancement, ECV = extracellular volume

**Fig. 5:**
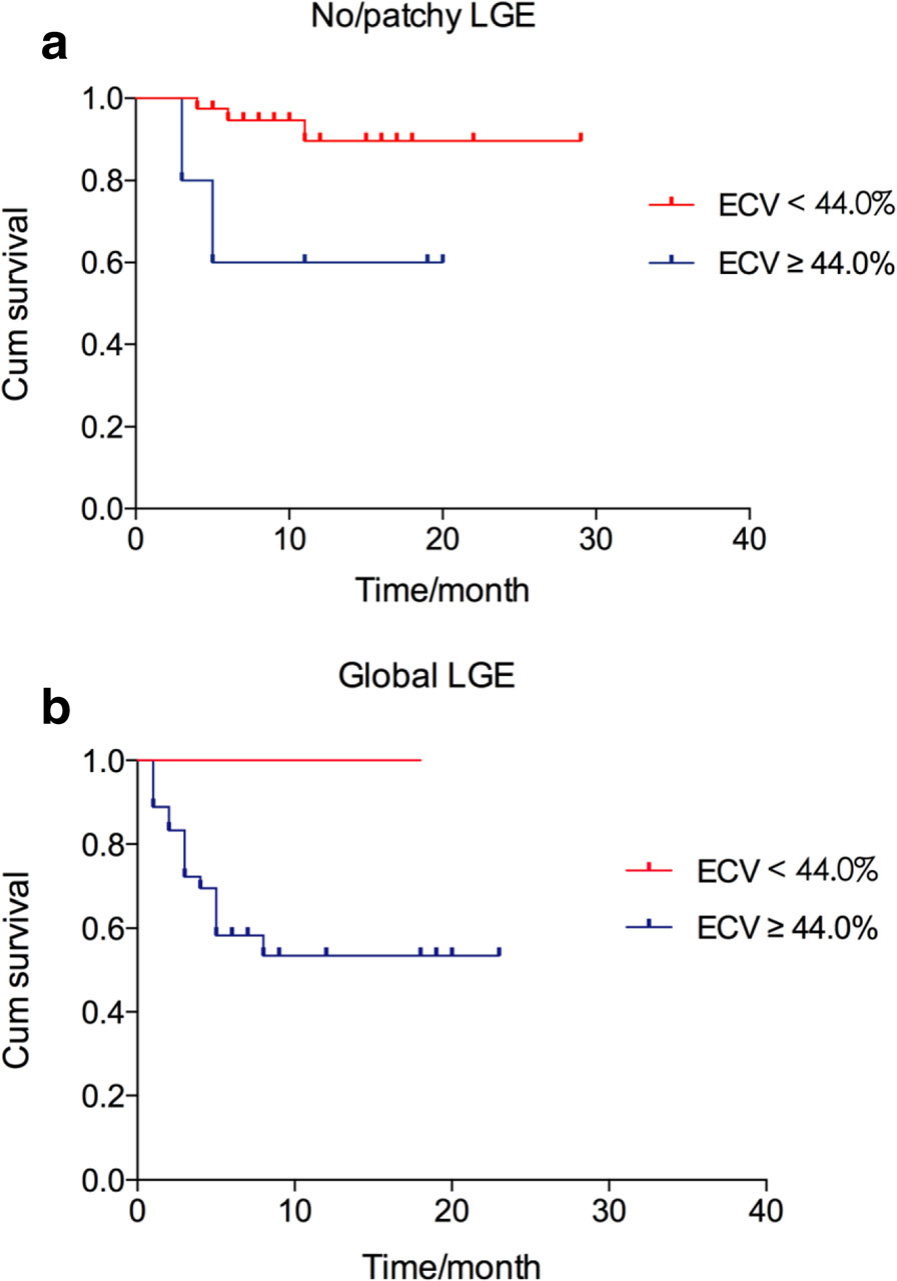
Kaplan-Meier survival curves for ECV in subgroups with the same LGE pattern. (**a**) In subgroups with no/patchy LGE (*n* = 44, ECV, 35.8 ± 6.8%), patients with ECV < 44.0% and ECV ≥ 44.0% differed significantly in survival probability (94.7% vs. 60.0% at the 8th month, log rank *P* = 0.029). (**b**) In subgroups with global LGE (*n* = 38, ECV, 53.4 ± 6.2%), patients with ECV < 44.0% had a survival probability of 100% and patients with ECV ≥ 44.0% had a survival probability of 53.5% at the 8th month. LGE = late gadolinium enhancement, ECV = extracellular volume

Univariate analysis showed that both ECV ≥ 44.0% (HR 7.677, 95% CI 2.256–26.128, *P* = 0.001) and global LGE (HR 5.047, 95% CI 1.971–12.926, P = 0.001) were significantly prognostic for mortality. Patients categorized by median native T1 (1456 ms) did not differ significantly in survival probability (Tarone-Ware *P* = 0.069) (Fig. 4-a). Patients categorized by median ECV (ECV < 44.0% and ECV ≥ 44.0%) differed significantly in survival probability (log rank P = 0.001) (Fig. 4-b). Patients with no or patchy LGE and global LGE differed significantly in survival probability (log rank P = 0.001) (Fig. 4-c). We categorized patients into different subgroups, one with global LGE (*n* = 38, ECV, 53.4 ± 6.2%) and the other with no/patchy LGE (*n* = 44, ECV, 35.83 ± 6.8%). In subgroups with the same LGE pattern, patients with ECV < 44.0% and ECV ≥ 44.0% differed significantly in survival probability (log rank *P* = 0.029), as shown in Fig. 5.

**Fig. 6:**
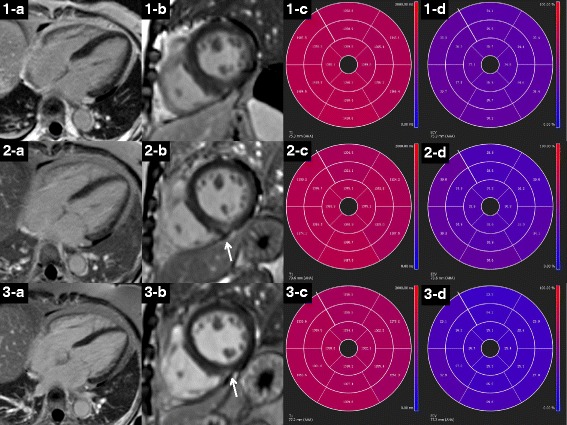
LGE images, native T1 and ECV bull’s eye plots of a 57-year-old female patient at baseline (1-a,b,c,d), 12-month (2-a,b,c,d) and 24-month (3-a,b,c,d) follow-up. At baseline, the patient showed no LGE (1-a, b) but elevated native T1 (1-c) and ECV (1-d) values. After chemo-therapy, the patient has a progressive decline in native T1 and ECV (at baseline, 12-month and 24-month follow-up: 1390 ms, 1371 ms, 1330 ms and 36.3%, 34.4%, 26.4%). At the 12-month follow-up, a new patch of mid-myocardial LGE appeared (2-b: arrow), which was not of typical position and pattern in AL amyloid, and seemed to regress at the 24-month follow-up. LGE = late gadolinium enhancement, ECV = extracellular volume

Eight patients (Mayo stage I/II/III/IV, 1/1/3/3; no/patchy/extensive LGE, 1/3/4) underwent follow-up CMR scans. The median interval between baseline and follow-up CMR scans was 12 months. All subjects completed a standard course of BCD chemotherapy and achieved a complete response (CR) or very good partial response (VGPR). For the patient with no LGE, the dynamic changes of LGE, native T1 and ECV are shown in Fig. 6. Another patient showed a significant regression of LGE as well as decreases of native T1 (1658 ms to 1490 ms) and ECV (62.7% to 51.4%). The other 6 patients showed no prominent progressions or regressions of LGE, and different trends of native T1 and ECV (increases in 2 patients, decreases in 2 patients, and no significant changes in 2 patients).

### Multivariate analysis

ECV and LGE were analyzed in separate multivariate Cox models because they had a correlation value ρ of 0.889. As shown in Table 2, for all AL amyloid patients, ECV ≥ 44.0% was significantly prognostic for mortality (HR 7.249, 95% CI 2.039–25.771, *P* = 0.002) in a multivariate Cox model correcting for age (HR 1.082, 95% CI 1.022–1.144, *P* = 0.006) and septal thickness (HR 1.175, 95% CI 1.035–1.335, *P* = 0.013). The Harrell’s C statistic was 0.62. Global LGE (HR 4.804, 95% CI 1.751–13.179, P = 0.002) was significantly prognostic for mortality in a multivariate Cox model correcting for NYHA (HR 2.253, 95% CI 1.385–3.666, *P* = 0.001) and septal thickness (HR 1.130, 95% CI 1.018–1.255, *P* = 0.022). The Harrell’s C statistic was 0.60.

Survival analysis separated by therapy status were performed. In the 71 patients without therapy at baseline, 52 (73%) patients were alive at the time of last follow-up. As shown in Table 5, ECV ≥ 44.0% (HR 4.599, 95% CI 1.493–14.165, *P* = 0.008) and global LGE (HR 4.442, 95% CI 1.578–12.389, *P* = 0.015) were independently prognostic for mortality, while median native T1 (1456 ms) was not prognostic for mortality (Tarone-Ware *P* = 0.108). In the 59 patients received therapy during the follow-up, 46 (78%) patients were alive. ECV ≥ 44.0% (HR 5.926, 95% CI 1.312–26.753, *P* = 0.021) and global LGE (HR 4.981, 95% CI 1.369–18.128, P = 0.015) were prognostic for mortality in univariate Cox model, but not prognostic in any multivariate Cox model. Median native T1 was not prognostic for mortality (Tarone-Ware *P* = 0.105).

**Table 5:** Univariate and multivariate Cox proportional hazard analysis in patients without therapy at baseline

	Univariate		Multivariate		Multivariate	
	HR (95% CI)	*P*	HR (95% CI)	*P*	HR (95% CI)	*P*
Age, per 1 year increase	1.053 (1.001–1.108)	0.046	1.067 (1.012–1.125)	0.011	1.060 (1.007–1.116)	0.025
NYHA	2.405 (1.450–3.988)	0.001	1.752 (1.010–3.039)	0.056	1.776 (0.924–3.414)	0.085
Mayo Stage	1.985 (1.212–3.252)	0.006	1.406 (0.736–2.683)	0.30	1.443 (0.779–2.672)	0.24
E/E’, per 1 unit increase	1.073 (1.003–1.114)	0.042	1.235 (0.477–3.134)	0.28	1.296 (0.463–3.188)	0.40
LVEF, per 1% increase	0.966 (0.939–0.994)	0.016	0.995 (0.960–1.033)	0.81	0.987 (0.954–1.021)	0.44
Septal thickness, per 1 mm increase	1.115 (1.020–1.219)	0.017	1.112 (0.988–1.251)	0.078	1.086 (0.973–1.213)	0.041
ECV ≥44.0%	4.751 (1.572–14.360)	0.006	4.599 (1.493–14.165)	0.008	–	–
Global LGE	4.041 (1.452–11.246)	0.007	–	–	4.442 (1.578–12.389)	0.015

All significantly prognostic factors in univariate analysis were listed. Univariate analysis was not performed for native T1 because the Kaplan-Meier curves crossed each other (Tarone-Ware, *P* = 0.069). Univariate analysis was not performed for log (cTnI) and log (NT-proBNP), as they were included in Mayo Stage. All clinically and statistically significant variates in univariate analysis were put into the multivariate Cox model. ECV and LGE were put in separate models because of a correlation ρ of 0.889. Backward regression was chosen. *HR* Hazard ratio, *CI* Confidence interval, *NYHA* New York Heart Association, *LVEF* Left ventricle ejection fraction, *LGE* Late gadolinium enhancement, *ECV* Extracellular volume

## Discussion

In this study, we examined the prognostic value of CMR ECV, LGE and native T1 in a Chinese population with AL amyloid. To the best of our knowledge, this is the first study to concurrently assess the prognostic value of T1 mapping parameters with LGE in AL amyloid. Our findings indicate that, while ECV and LGE functioned as independent prognostic factors for mortality in AL amyloid patients, native T1 did not display prognostic value. We also showed that in subgroups with the same LGE pattern, ECV remained prognostic.

We found AL amyloid patients with no LGE demonstrated increased native T1 and ECV, highlighting the importance of native T1 and ECV over LGE in early detection of myocardial involvement in this disorder. In agreement with other studies [11, 12], we showed that global LGE prognostic for mortality. We have also included a novel finding that subgroups with the same LGE pattern displayed ECV as a significant prognostic factor. LGE is the classic and most commonly performed CMR protocol for myocardial tissue characterization, and a typical pattern of global LGE serves as both diagnostic marker for cardiac AL amyloid and a prognostic marker for mortality [11–14]. However, early identification of mild cases of cardiac AL amyloid and other diffuse infiltrative cardiomyopathies are easily missed, [15, 16] since the basis of LGE lesion identification involves demarcating the abnormal tissue amidst normal tissue. It is better to perform T1 mapping scanning together with LGE scanning in AL amyloid patients, for native T1 and ECV provide additional diagnostic and prognostic information.

The current study is also the second overall study that focuses on the prognostic value of T1 mapping parameters in AL amyloid. A previous study demonstrated the prognostic value of native T1 and ECV for mortality using a 1.5 T scanner with a shMOLLI sequence [25], but LGE was not assessed. In this study, using a 3 T scanner with a MOLLI sequence and found that, regardless of disease course and therapy status, ECV was an independently prognostic factor for mortality with a similar cut-off value as the previous study.

Moreover, we also found that native T1 did not act as a prognostic factor, which is controversial with previous study. Previous studies have shown variations in ECV values using different scanning sequences including MOLLI and shMOLLI [24], and variations in native T1 values with different equipment manufacturers, scanning sequences and undefined physiological status of the patients [21–23]. Despite the emerging importance of T1 mapping, one fundamental issue to be solved is the evidence of a good reproducibility among different institutions. Combining other studies with our current study, we have shown that ECV is a better T1 mapping parameter and, as of now, cannot be replaced by native T1.

ECV calculation requires the administration of an IV contrast agent. However, renal function impairment is often seen in AL amyloid patients, since the kidney is one of the most commonly involved organs [1, 2]. This and other contraindications for the application of contrast agents may limit the use of ECV in this population. In this situation native T1 in combination with LVEF and inter-ventricular septum thickness seems to be the second best approach to detect diffuse myocardial involvement. Our data show that native T1 is not as prognostic as ECV, but still more sensitive than LGE for myocardial involvement in AL amyloid.

Our study has several limitations. One is the short follow-up with a median time of 8 months and a relatively low event proportion of 25.6%. Another limitation is that we do not have additional parameters to fully characterize the diastolic function. The third limitation is about the therapy status of the patients at baseline and during the follow-up, given the cardiotoxic effects of chemotherapy agents may be confounding factors. Besides, we found increased native T1 and ECV values in patients with no LGE, but only one such patient underwent myocardial biopsy verifying the result. Thus ours, like most studies in this area, suffer from the lack of diagnostic pathology.

## Conclusion

For myocardial tissue characterization, while LGE is a classic and most commonly performed parameter, ECV is a recently developed quantitative CMR parameter. The current study is the first to compare the prognostic value of T1 mapping parameters with LGE in AL amyloid. During a short follow-up interval, we showed that both ECV and LGE were promising prognostic factors for mortality in AL amyloid. Further, in patients with the same LGE pattern, ECV remained prognostic, suggesting the merit of using T1 mapping scanning in conjunction with LGE in this population. Native T1, however, was found to be not as equally prognostic as ECV or LGE. Thus, for suspected AL amyloid patients without contraindications, it is better to perform contrast enhancement scanning.

## Abbreviations

2D: Two-dimensional
AHA: American Heart Association
AL amyloidosis: Immunoglobulin light chain type amyloidosis
ANOVA: One-way analysis of variance
ASCT: Autologous stem cell transplant
BCD: Bortezomib, cyclophosphamide and dexamethasone
bSSFP: Balanced steady-state free precession
CI: Confidence intervals
CMR: Cardiovascular magnetic resonance
cTnI: Cardiac Troponin I
dFLC: Serum immunoglobulin free light chain difference
ECG: Electrocardiogram
ECV: Extracellular volume fraction
FA: Flip angle
HR: Hazard ratios
LGE: Late gadolinium enhancement
LV: Left ventricle/left ventricular
LVEDVi: LEFT ventricle end-diastolic volume index
LVEF: Left ventricular ejection fraction
MOLLI: Modified Look-Lockers Inversion-recovery
NT-proBNP: N-terminal pro-B-type natriuretic peptide
PSIR: Phase-sensitive inversion-recovery
shMOLLI: Shortened Modified Look-Lockers Inversion-recovery
TCD: Thalidomide, cyclophosphamide and dexamethasone
TE: Echo time
TR: Repetition time
TTE: Transthoracic echocardiography
